# Current state and potential of hospitals for automated healthcare-associated infection surveillance: data from 24 European countries, 2022 to 2023

**DOI:** 10.2807/1560-7917.ES.2026.31.19.2500736

**Published:** 2026-05-14

**Authors:** Ferenc Darius Rüther, Mariana Guedes, Elisabeth Presterl, Carl Suetens, Diamantis Plachouras, Maaike SM van Mourik, Stephanie van Rooden, ECDC PPS study group on automated surveillance of HAIs, Collaborators, Seven Johannes Sam Aghdassi, Lucie Bareková, Zrinka Bošnjak, Mireia Cantero, Elina Dobreva, Ólafur Guðlaugsson, Irena Klavs, Katrien Latour, Slavka Litvová, Stephen Murchan, Alexandre Mzabi, Dulce Pascoalinho, Radica Raičević, Elizabeth Anne Scicluna

**Affiliations:** 1 Charité – Universitätsmedizin Berlin, corporate member of Freie Universität Berlin and Humboldt-Universität zu Berlin, Institute of Hygiene and Environmental Medicine, Berlin, Germany; 2 National Reference Center for Surveillance of Nosocomial Infections, Berlin, Germany; 3 Infectious Diseases and Microbiology Division, Hospital Universitario Virgen Macarena, Seville, Spain; 4 Department of Medicine, University of Sevilla/Instituto de Biomedicina de Sevilla (IBiS)/Consejo Superior de Investigaciones Científicas (CSIC), Seville, Spain; 5 Department of Hospital Epidemiology and Infection Control, Medical University of Vienna, Vienna, Austria; 6 European Centre for Disease Prevention and Control, Stockholm, Sweden; 7 Department of Medical Microbiology and Infection Control, University Medical Centre Utrecht, Utrecht, the Netherlands; 8 Department of Epidemiology and Surveillance, Centre for Infectious Disease Epidemiology and Surveillance, National Institute for Public Health and the Environment (RIVM), Bilthoven, the Netherlands; 9 The members of the study group are listed under Collaborators and at the end of the article.

**Keywords:** healthcare-associated infections, automated surveillance, point prevalence survey, infection prevention and control

## Abstract

**BACKGROUND:**

Although electronic health records are increasingly used for automated surveillance (AS) of healthcare-associated infections (HAIs), implementation is still a challenge. To develop more targeted implementation initiatives across Europe, knowledge about the current state of AS and potential to implement AS systems is needed.

**AIM:**

To assess the adoption and feasibility of AS based on the 2022–2023 European Centre for Disease Prevention and Control (ECDC) Point Prevalence Survey (PPS).

**METHODS:**

The 2022–2023 ECDC PPS included questions on the degree of AS and digital data storage for seven HAIs. Descriptive analyses of the responses were performed and stratified by geographic region and hospital characteristics. Categorical variables were analysed as such and converted to ordinal scales.

**RESULTS:**

Overall, 992 hospitals from 24 European countries participated. Across all seven HAIs, fully manual surveillance was the most common method (from healthcare-associated pneumonia (HAP) 38.8% to *Clostridioides difficile* infection (CDI) 45.4%). A considerable proportion, i.e. 19.3% (HAP) to 29.8% (CDI), employed some form of automation (automated denominator 5.3–11.3%; semi-automated 12.2–16.9%; fully automated 1.8–2.9%). Many hospitals not employing AS had required source data digitally stored. Generally, tertiary hospitals had higher levels of automation and digital data storage compared with other hospital types. Smaller hospitals (≤ 250 beds) had lower levels of automation, but a similar level of digital data storage compared with larger hospitals.

**CONCLUSION:**

This study highlights variability in AS implementation and digital potential across European hospitals and underscores the need for targeted strategies to advance AS adoption and optimise surveillance.

**Key public health message**

**What did you want to address in this study and why?**

This study aims to understand the state and potential for automated surveillance (AS) of healthcare-associated infections in hospitals across Europe. Automation of surveillance, based on digital routine care data, allows the monitoring of these types of infections with fewer resources. With less capacity needed for surveillance, more types of infections can be monitored continuously, which can help prevent infections and improve patient safety.

**What have we learnt from this study?**

By analysing data from a survey on AS from 24 European countries, we found that time-consuming fully manual surveillance is still the most common approach (mean of infection types: 42.4%), although many hospitals already have essential digital data to support some automation. This shows a clear opportunity to expand AS using existing resources, especially (but not only) in larger and tertiary hospitals that are further along in digital readiness.

**What are the implications of your findings for public health?**

European initiatives and incentives tailored to current level of digitalisation of data and AS will improve and expand automation in monitoring healthcare-associated infections. Supporting hospitals in adopting these systems can strengthen infection prevention and overall healthcare quality across Europe.

## Introduction

Healthcare-associated infections (HAIs) considerably impact morbidity, mortality and healthcare costs [1]. Despite an increase in evidence-based intervention strategies for HAIs [2], these types of infections remain prevalent, with estimated 8.9 million HAI episodes per year in the European Union/European Economic Area (EU/EEA) [3].

Surveillance of HAIs comprises continuous, systematic collection and analysis of health-related data using reproducible and objective definitions, and consolidation and dissemination of the data to evaluate temporal changes and the effect of interventions [4,5]. This method is well-established to reduce HAI rates and is a cornerstone of infection prevention and control (IPC) by identifying areas for prevention and evaluating the effect of interventions [6-9].

With the increasing adoption of electronic health records (EHR), ongoing efforts have been directed towards development of automated surveillance (AS) methods to monitor HAIs. This transition likely leads to more standardised surveillance and more timely feedback, which enhances the ability to efficiently monitor the rates of multiple types of infections simultaneously [4,10] without adding to the workload of the infection control practitioner, although requiring investments from information technology (IT) staff [11].

Automated surveillance can replace different steps of the manual surveillance process, including automating the selection of the surveillance population and classification of patients as having an HAI or not, with a manual confirmation step (semi-AS) or without manual confirmation (fully AS) [12]. Although EHR have been increasingly adopted across Europe, solid implementation of AS including collection and integration of digital data sources remains challenging. Hospital-specific configuration of EHR systems demands local IT expertise, and capacity for validation and maintenance for automated data collection (including funding), although these resources are not always available. In addition, for some surveillance targets, unstructured data, e.g. free text notes not standardised to (international) terminology, are essential source data, further complicating implementation [4,13,14]. However, since 2015 several surveillance networks have implemented AS systems of various HAIs [15-19] and in 2022 the European Centre for Disease Prevention and Control (ECDC) launched an initiative to explore possibilities to automate surveillance [20,21]. Many more initiatives have been developed and implemented in individual hospitals, with the majority being unpublished [22,23]. In order to develop strategies for large-scale implementation and harmonisation of AS initiatives, better insight into these initiatives is needed. In addition, assessing the potential for automation — based on the availability of digital source data essential for denominator selection or HAI identification — represents an important starting point.

Since 2011, the ECDC periodically performs European Point Prevalence Surveys (PPS) of HAIs and antimicrobial use (AMU) in European acute care hospitals [24-26]. The protocols of these surveys have been adapted to the experiences and needs identified from previous PPS results. As the current degree of digitalisation and automation of HAI surveillance has not been systematically assessed across Europe, the 2022–2023 ECDC PPS presented a unique opportunity to learn about the current state and potential of hospitals throughout Europe for automated HAI surveillance.

Based on the data collected in the 2022–2023 ECDC PPS, this study aims to describe the current degree of AS of HAIs and digital data storage at the European level. It also seeks to explore relations with geographic regions and hospital structure (ownership, type, size) that could facilitate or hinder its implementation.

## Methods

### Study design

This study is a secondary, cross-sectional analysis of data collected within the framework of the 2022–2023 ECDC PPS. The PPS itself was conducted according to the standard ECDC protocol [27], which contained additional questions (‘AS questions’) on the implementation of automated surveillance. Countries were not obligated to incorporate AS questions into their national PPS protocols and adaptations on a national level were possible, allowing flexibility based on local priorities and resources. The ECDC PPS protocol [27] was disseminated electronically to departments of infection control in participating acute care hospitals in each country, coordinated through national contact points and ECDC channels, to ensure widespread participation and consistency in data collection.

### Study population and data collection

All data for the present study were from 2022–2023 ECDC PPS database, provided by Austria, Belgium, Bulgaria, Croatia, Cyprus, Czechia, Estonia, Finland, France, Germany, Greece, Hungary, Iceland, Ireland, Italy, Kosovo^‡^, Latvia, Lithuania, Luxembourg, Malta, Montenegro, the Netherlands, Norway, Poland, Portugal, Romania, Serbia, Slovakia, Slovenia, Spain and Sweden. Data were shared by ECDC in accordance with ECDC’s policy on data submission, access and use of data within the European surveillance portal for infectious diseases [28].

Data from a total of 1,623 hospitals, corresponding to approximately 11.4% of all European acute care hospitals, were submitted to 2022–2023 ECDC PPS, with 1,332 hospitals included in the final ECDC sample for analysis [26]. Following the data collection phase, a subset of PPS data specifically relevant to the AS questions (including hospital characteristics such as hospital size, hospital type and hospital ownership) was retrieved from the 2022–2023 ECDC PPS database.

### Automated surveillance questions

The AS questions were proposed by the PRAISE (Providing a Roadmap for Automated Infection Surveillance in Europe) network and were included in the survey in a standardised format with a combination of multiple-choice and categorical response options to allow consistent cross-country comparisons [12]. Two groups of questions were included in the protocol to assess: (i) Current degree of automation of surveillance for seven different HAIs i.e. healthcare-associated bloodstream infection (HA-BSI), catheter-associated urinary tract infection (CAUTI), *Clostridioides difficile* infection (CDI), central line-associated bloodstream infection (CLABSI), healthcare-associated pneumonia (HAP), surgical site infection (SSI) and ventilator-associated pneumonia (VAP). Hospitals performing surveillance for these HAIs could indicate the degree of automation by choosing between fully manual, automated denominator (i.e. the selection of all patients, procedures, or devices under surveillance), semi-automated or fully automated surveillance, or ‘other’. The meaning of ‘Other’ was defined in the PPS protocol as the use of electronically available databases to preselect patients for inclusion in surveillance and/or to identify patients requiring manual confirmation of HAI presence, without automated direct linkage to an electronic surveillance record, i.e. still requiring manual steps. In this study, it was interpreted as a non-specified form of surveillance, for example when it was partially automated in some areas of the hospital but overall conducted manually (more details included in Supplementary Material S1 and S2). (ii) Feasibility of automated HAI surveillance. The protocol examined whether key data elements for automation of HAI surveillance were stored digitally and, if so, whether they were organised in a structured format, including: admission and discharge dates at hospital level, admission and discharge dates at unit level, use of central lines (date of insertion/removal and type), use of mechanical ventilation or intubation (date of insertion/removal), surgical procedures (such as procedure codes or date of surgery), use of urinary catheters (date of insertion/removal), microbiology culture results and antimicrobial prescriptions. A full list of AS questions with definitions of the response options is provided in Supplementary Material S1.

### Data handling

On submission of the completed survey to ECDC, additional data validation or systematic quality checks specific to the AS questions were not performed. Missing values for the AS questions were permitted and could arise if a participating hospital skipped one or more AS questions during survey completion. In addition, missing values were recorded for hospitals participating in the PPS when countries did not include the AS questions in their local PPS protocol at all. In the dataset, all missing values were coded as ‘unknown’. As ‘unknown’ was also explicitly offered as a response option for questions on the digital storage of key data elements, it was not possible to distinguish between true non-response and an explicit ‘unknown’ answer for these items (Supplementary Material S1). Consequently, data fields to which ‘unknown’ was assigned were neither excluded nor imputed, but were treated as a separate category in the analyses.

### Country and hospital selection

Seven countries that did not include the AS questions in their national PPS protocol (France, Italy, Latvia, Lithuania, the Netherlands, Norway and Sweden), were excluded from further analysis. Thus, response rates for the AS questions were only calculated among hospitals from countries that implemented the AS questions and cannot be extrapolated to all hospitals invited to participate in the PPS. Also, hospitals with non-response were excluded, which was assumed when all AS question data fields contained the entry ‘unknown’. The remaining hospitals were included in the final data analysis.

### Data annotation and level construction

The raw data export was annotated based on geographic region and level of automation/digital storage as follows: Countries participating in the PPS were assigned to one of the four geographic regions (eastern, northern, southern, western) in Europe as defined by the United Nations M49 standard country or area codes for statistical use [29]. Analyses with regard to the location of hospitals were conducted on an aggregated level of these regions to prevent potential identification of individual hospitals or countries. 

Based on responses to the two topics of AS questions (the current degree of automation of surveillance of HAIs and the feasibility of automated HAI surveillance), we composed two distinct 'levels' to facilitate comparability across hospitals: (i) level of automation of HAI surveillance (based on seven questions for specific types of HAIs), (ii) level of digital storage of key data elements for automation of HAI surveillance (based on eight underlying questions for specific data elements). The levels were calculated for all responding hospitals by adding up for all questions of one topic and normalised to a 0–100 scale, as described in more detail in Supplementary Material S2.

### Statistical analysis

A descriptive data analysis was performed for the overall cohort of hospitals and for hospitals stratified by region, focusing on hospital characteristics and responses to the AS questions. To assess the plausibility of responses and the feasibility of AS for a selection of HAIs (HA-BSI, CAUTI, CLABSI, SSI, VAP), the reported degree of automation was compared with the digital storage of data, for which specific key data elements are essential for automation, depending on the type of HAI (i.e. microbiology results, surgical procedure codes, use of central lines, use of mechanical ventilation, use of urinary catheters) by using stacked bar charts. The median and interquartile ranges (IQR) of the level of automation and digital storage of key data elements were calculated in total and stratified by geographic region, hospital ownership, hospital type and hospital size. In a sensitivity analysis, hospitals responding to all AS questions were compared with hospitals with at least one response stored as unknown. The latter group mainly represented hospitals with an incomplete response but also contained hospitals that explicitly answered with unknown to at least one of the AS questions on the digital storage of data. For the calculation of complete response rates, all questionnaires with at least one response recorded as unknown were considered incomplete.

Parameters were assessed for relevant differences by the chi-square test (for categorical variables), the Mann–Whitney U test (for continuous variables comparing two groups) or the Kruskal-Wallis test with Dunn-Bonferroni post-hoc analysis (for continuous variables comparing more than two groups). The latter considers the relative ranks of each observation, not just medians. Differences in the spread, shape or presence of outliers can cause statistically significant differences despite identical medians. Collinearity of hospital characteristics was assessed by generating crosstabs and calculating Cramer’s V for hospital size, type, ownership and region. For all statistical procedures, a p-value < 0.05 defined significance. All analyses were performed with SPSS version 29.0 (IBM Corp.).

## Results

**Figure 1:**
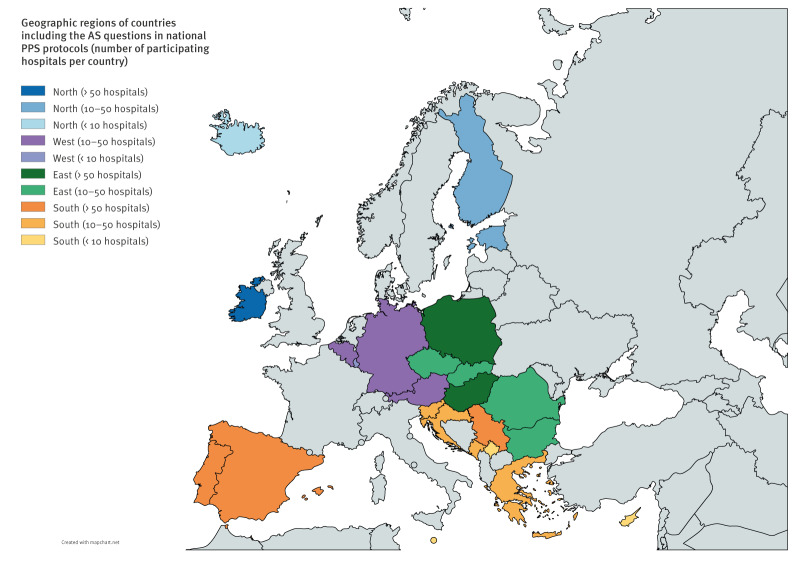
Participating countries including questions on automated surveillance of healthcare-associated infections in their national protocols of the ECDC point prevalence survey, 2022–2023 (n = 24)

AS: automated surveillance; ECDC: European Centre for Disease Prevention and Control; PPS: point prevalence survey.

Overall, 24 of 31 countries incorporated the AS questions into their local PPS protocols (Figure 1), covering 1,040 of the 1,332 participating hospitals (78.1%) of the final 2022–2023 ECDC PPS sample [26].

Countries are colour coded according to geographic region as per the United Nations M49 standard. Colour shades indicate categories of absolute numbers of hospitals participating.

**Figure 2:**
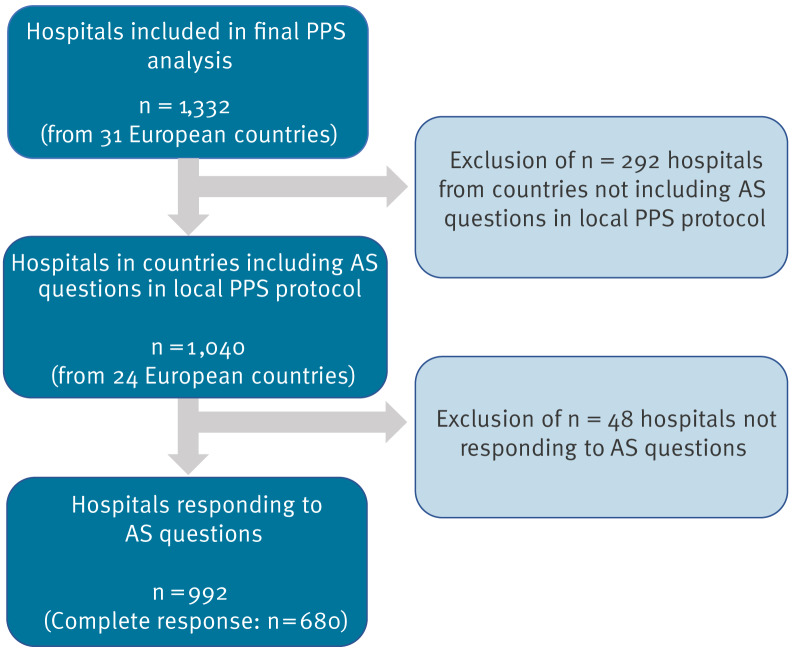
Flowchart of acute care hospitals included in this study on automated surveillance of healthcare-associated infections, 24 European countries, 2022–2023 (n = 992 hospitals)

AS: automated surveillance; ECDC: European Centre for Disease Prevention and Control; PPS: point prevalence survey.

Of these, 992 hospitals from 24 countries responded to at least one AS question (95.4% response rate, 74.5% of the 2022–2023 ECDC PPS sample) and were included for further analyses; 680 hospitals from 22 countries responded to all AS questions (65.4% completeness) (Figure 2).

Countries participating in the ECDC PPS survey with hospitals responding to AS questions: Austria, Belgium, Bulgaria, Croatia, Cyprus, Czechia, Estonia, Finland, Germany, Greece, Hungary, Iceland, Ireland, Kosovo^‡^, Luxembourg, Malta, Montenegro, Poland, Portugal, Romania, Serbia, Slovakia, Slovenia and Spain.

The majority of the 992 responding hospitals were located in the geographic South (41.0%) and East (32.1%). The most common category of hospital ownership was public (76.5%), and the majority had a secondary level of care (38.6%) (Table 1). Hospital characteristics stratified by geographic region are provided in Supplementary Table S1. When looking at collinearity of hospital characteristics, tertiary-level hospitals tended to be larger than other hospital types (data not shown).

**Table 1:** Descriptive analysis of acute care hospitals responding to the questions on automated surveillance of healthcare-associated infections, 24 European countries, 2022–2023 (n = 992 hospitals)

Hospital characteristics	Number of hospitals	%
Geographic region		
East (n = 6 countries)	318	32.1
North (n = 4 countries)	124	12.5
South (n = 10 countries)	407	41.0
West (n = 4 countries)	143	14.4
Hospital ownership		
Public	759	76.5
Private for-profit	121	12.2
Private not-for-profit	84	8.5
Other/unknown	28	2.8
Hospital type		
Primary level (district hospital or first-level referral)	250	25.2
Secondary level (provincial hospital)	383	38.6
Tertiary level (regional or tertiary-level hospital)	226	22.8
Specialised	131	13.2
Unknown	2	0.2
Hospital capacity, median (IQR)		
Number of hospital beds	270 (138–493)	
Number of ICU beds^a^	10 (5–23)	
Number of patient days per year^b^	59,479 (27,759–106,672)	

ICU: intensive care unit; IQR: interquartile range.

^a^ n = 977 hospitals.

^b^ n = 968 hospitals.

Other or unknown: Hospital ownership cannot be categorised as one of one of the above, or hospital ownership is unknown.

In the following, results are presented primarily by type of HAI, with analyses by geographic region and hospital characteristics provided in the Supplementary Materials for reference.

### Reported degree of automation of surveillance of different healthcare-associated infections 

The aggregated response patterns of hospitals regarding the reported degree of automated surveillance by HAI type are shown in Table 2. The implementation of surveillance (regardless of automation) was most frequently reported for CDI (85.8%), HA-BSI (83.5%) and CLABSI (82.7%). For all HAIs that were part of the survey, the largest proportion of hospitals reported conducting fully manual surveillance (range: 38.8–45.4). Automation of surveillance limited to the collection of denominator data was reported by 5.3% (HAP) to 11.3% (CDI) of hospitals (Table 2).

**Table 2:** Degree of automated surveillance of acute care hospitals per type of healthcare-associated infection, 24 European countries, 2022–2023 (n = 992 hospitals)

Degree of automated surveillance^a^	Type of healthcare-associated infection													
	HA-BSI		CAUTI		CDI		CLABSI		HAP		SSI		VAP	
	n	%	n	%	n	%	n	%	n	%	n	%	n	%
No response or unknown	8	0.8	9	0.9	10	1.0	10	1.0	15	1.5	10	1.0	16	1.6
Not performed	155	15.6	245	24.7	131	13.2	162	16.3	314	31.7	210	21.2	285	28.7
Other**^b^**	105	10.6	92	9.3	105	10.6	104	10.5	86	8.7	87	8.8	89	9.0
Fully manual	433	43.6	419	42.2	450	45.4	448	45.2	385	38.8	422	42.5	391	39.4
Automated denominator	100	10.1	60	6.0	112	11.3	89	9.0	53	5.3	88	8.9	57	5.7
Semi-automated	168	16.9	147	14.8	155	15.6	155	15.6	121	12.2	156	15.7	135	13.6
Fully automated	23	2.3	20	2.0	29	2.9	24	2.4	18	1.8	19	1.9	19	1.9
**Any type of automation^c^**	**291**	**29.3**	**227**	**22.8**	**296**	**29.8**	**268**	**27.0**	**192**	**19.3**	**263**	**26.5**	**211**	**21.2**

CAUTI: catheter-associated urinary tract infection; CDI: *Clostridioides difficile* infection; CLABSI: central line-associated bloodstream infection; HA-BSI: healthcare-associated bloodstream infection; HAP: healthcare-associated pneumonia; SSI: surgical site infection; VAP: ventilator-associated pneumonia.

**^a^** The response options for automated surveillance questions are ordered by increasing degree of automation.

**^b^** The meaning of ‘Other’ was interpreted as a form of surveillance, but deemed insufficient to be classified within any of the other categories listed in the table. In the PPS protocol, ‘Other’ was defined as the use of electronically available databases to preselect patients for inclusion in surveillance and/or to identify patients requiring manual confirmation of HAI presence, without automated direct linkage to an electronic surveillance record, i.e. still requiring manual steps. More details are provided in Supplementary Material S1 and S2. In practice, it is likely that hospitals also selected ‘Other’ in situations not strictly covered by the protocol definition, for example, when a particular form of surveillance was partially automated in some areas of the hospital but still conducted manually in others. Therefore, the ‘Other’ category is used to indicate that a non-specified form of surveillance is implemented in parts of the hospital, without further classification.

**^c^** ‘Any type of automation’ includes automated denominator, semi-automated or fully automated.

While 12.2% (HAP) to 16.9% (HA-BSI) of hospitals reported to perform semi-automated surveillance, 1.8% (HAP) to 2.9% (CDI) indicated to conduct fully AS (Table 2). Twelve hospitals reported fully AS for all considered HAI. Data on the geographical distribution can be found in Supplementary Figure S1.

### Feasibility of automated healthcare-associated infection surveillance

Considering the feasibility of automated HAI surveillance, responses of hospitals to AS questions about data storage of key data elements are presented in Table 3. Key data elements for AS, with the highest proportions of hospitals indicating a hospital-wide digital storage of data, were admission and discharge dates at hospital and ward level (76.4% and 75.0%, respectively) and microbiology results (70.1%), while notably fewer hospitals reported a digital storage of data on the use of urinary catheters (38.9%), central lines (37.3%) or mechanical ventilation (31.3%) or (Table 3). Similarly, a structured and well-defined storage of data was most often reported for admission and discharge dates and for microbiology results (Table 3).

**Table 3:** Feasibility of automated surveillance of healthcare-associated infections regarding different key data elements for automation in acute care hospitals, 24 European countries, 2022–2023 (n = 992 hospitals)

Key data elements	Admission and discharge dates				Antimicrobial prescriptions		Central lines		Microbiology results		Mechanical ventilation		Surgical procedures		Urinary catheters	
	Hospital level		Ward level													
	n	%	n	%	n	%	n	%	n	%	n	%	n	%	n	%
**Data are digitally stored**																
No response or unknown	77	7.8	78	7.9	132	13.3	86	8.7	77	7.8	114	11.5	93	9.4	92	9.3
No	100	10.1	103	10.4	314	31.7	384	38.7	145	14.6	392	39.5	198	20.0	397	40.0
Yes, in specific wards	57	5.7	67	6.8	79	8.0	152	15.3	75	7.6	176	17.7	122	12.3	117	11.8
Yes, hospital-wide	758	76.4	744	75.0	467	47.1	370	37.3	695	70.1	310	31.3	579	58.4	386	38.9
**Data are stored structured and well-defined**																
No response or unknown	156	15.7	156	15.7	195	19.7	174	17.5	154	15.5	173	17.4	171	17.2	168	16.9
Not applicable	38	3.8	39	3.9	146	14.7	172	17.3	68	6.9	205	20.7	78	7.9	171	17.2
No	98	9.9	105	10.6	225	22.7	277	27.9	151	15.2	259	26.1	173	17.4	288	29.0
Yes	700	70.6	692	69.8	426	42.9	369	37.2	619	62.4	355	35.8	570	57.5	365	36.8

The stratification of results by geographic region revealed slight differences between the regions but not a distinct pattern. Details on the geographical distribution can be found in Supplementary Figures S2 and S3.

### Aligning the reported degree of automation of healthcare-associated infection surveillance to the feasibility of automated HAI surveillance

To further investigate the potential for automation (e.g. hospitals that reported a lower degree of automation), and to identify discordant response patterns (e.g. hospitals indicating full automation but no digital storage of the required data), we visually aligned the reported degree of automated surveillance per HAI with its feasibility, defined as the digital storage of required data elements. For each HAI, around 80% of all hospitals that indicated to perform some degree of AS (i.e. automated denominator, semi- or fully AS) reported to have the corresponding key data element digitally stored. The full analysis is provided in Supplementary Figure S4.

Considering only hospitals employing fully AS for HA-BSI (n = 23), CAUTI (n = 20), CLABSI (n = 24), SSI (n = 19) and VAP (n = 19), few hospitals still responded to have no digital storage of the respective key data elements (HA-BSI: n = 1, CAUTI: n = 3, CLABSI: n = 2, SSI: n = 2 and VAP: n = 1) or the stored response was ‘Unknown’ (HA-BSI: n = 3, CAUTI: n = 4, CLABSI: n = 4, SSI: n = 3 and VAP: n = 4). Of the twelve hospitals that reported full AS for all HAIs considered, five hospitals indicated no digital storage of the key data elements, or the response was unknown or missing.

In hospitals that did not perform surveillance of HAIs or conducted HAI surveillance in a fully manual manner, digital storage of the corresponding key data was less frequent than in hospitals with some kind of AS. Still, a considerable proportion of hospitals digitally stored certain key data elements such as microbiology results or surgical procedures while not (yet) employing a system for AS of HA-BSI or SSI (Supplementary Figure S4A and S4D). The results stratified by geographic regions are depicted in Supplementary Figure S5.

### Current level of automation of healthcare-associated infection surveillance and digital storage of data

**Figure 3:**
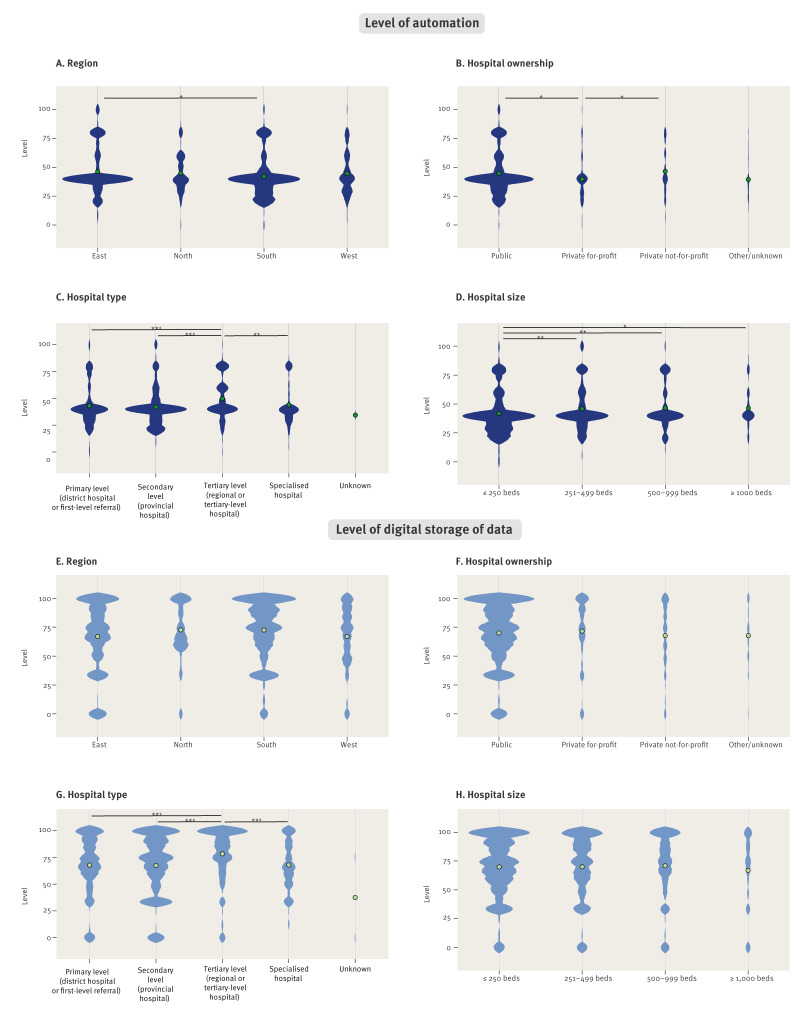
Levels of automated surveillance of healthcare-associated infections and digital storage of key data elements, stratified by hospital characteristics, 24 European countries, 2022–2023 (n = 992 hospitals)

The overall median level of automated HAI surveillance, calculated for every hospital by combining the responses to the different HAIs as described in Supplementary Material S2, was 40.0 (IQR: 34.3–48.6) out of 100 and the median level of digital storage of key data elements for AS was 75.0 (IQR: 58.3–91.7). As these levels allow assessment of distributions, violin plots were generated to visualise the corresponding density curves, stratified by different hospital characteristics (Figure 3).

A–D: Level of automation stratified by region (A), hospital ownership (B), hospital type (C) and hospital size (D).

E–H: Level of digital storage of data stratified by region (E), hospital ownership (F), hospital type (G) and hospital size (H).

Calculation of levels is described in Supplementary Material S2. The violin plots depict the distribution of levels using density curves. The width of each curve illustrates the number of hospitals per data point. The green dots represent mean values, asterisks represent p values below 0.05 (*: < 0.05, **: < 0.01, ***: < 0.001) in the post-hoc Dunn-Bonferroni test comparing weighted ranks.

Stratified by geographic region, the distribution of levels of automation differed slightly between eastern and southern hospitals. No relevant differences were found for the level of digital storage of data (Figure 3A and 3E, Supplementary Table S2).

Regarding hospital ownership, the level of automation appeared slightly higher in public and private not-for-profit hospitals compared with private-for-profit hospitals. There was no difference in the level of digital storage of data for the different types of ownership (Figure 3B and 3F, Supplementary Table S2). Hospitals with a tertiary level of care had both higher levels of automation and higher levels of digital storage of data compared with primary care, secondary care and specialised hospitals (Figure 3C and 3G, Supplementary Table S2). Regarding hospital size, hospitals with ≤ 250 beds had slightly lower levels of automation than larger hospitals, while the level of digital storage of source data showed no differences (Figure 3D and 3H, Supplementary Table S2).

### Hospitals with incomplete response to all automated surveillance questions 

A comparison of hospitals responding to all AS questions with hospitals responding incompletely is provided in Supplementary Table S3 and Supplementary Table S4. In a sensitivity analysis, no relevant differences were determined between hospitals with complete responses to AS questions compared with hospitals with at least one missing or unknown response (Supplementary Table S3).

## Discussion

Automated surveillance based on available well-defined, structured and robust data will modernise surveillance of HAIs and substantially improve the quality of healthcare. It has the potential to open a new chapter for evidence in HAI prevention in any medical specialty. A roadmap has been developed which identified important factors to advance the operationalisation of automated surveillance [12]. We provide an overview of the preparedness levels for automation in hospitals from all over Europe using data of ECDC’s PPS of HAIs and AMU in European acute care hospitals in 2022–2023 [26]. While the findings suggest that HAI surveillance is still predominantly manual, a considerable number of hospitals reported performing some kind of AS for the most common HAIs, either using automated denominators, semi- or even fully AS. Furthermore, key data elements essential to implement AS are available as digital and structured data in a considerable portion of hospitals, particularly in hospitals already doing HAI surveillance, both manually or with some degree of automation.

Hospitals from countries in the northern and western regions of Europe reported higher level of automation, which may reflect a longer experience or a higher investment into surveillance systems. Hospitals with ≥ 250 beds and tertiary care level have higher automation levels, when compared with hospitals of other categories, which may reflect the recognised advantage of AS when having many and complex patients. Moreover, tertiary care level hospitals have higher digital data storage levels, probably demonstrating the supportive digital infrastructure units available in these hospitals for administration, EHR and clinical research, since they are usually associated with universities. The higher level of automation in larger and tertiary care hospitals may reflect the fact that these hospitals usually have more resources, more mature data governance and increased IT capacity. Although EHR are frequently customised to support the process of care, their standardisation and interoperability for secondary use of data may not be a priority. However, the observation that data digitalisation was reported regardless of hospital size supports the notion that the data infrastructure necessary to implement AS may already be available.

Our study demonstrates that hospitals in Europe can be classified in two groups: hospitals already familiar with some kind of AS and digitalised data (primarily public and private not-for-profit, tertiary care, ≥ 250 beds, northern and western countries), and hospitals where surveillance is mostly done manually or not performed at all and data are only partially digitalised. Future action plans for AS should be tailored to this distinction: while the first group will benefit most from guidance for the implementation of automated systems, the second group needs to first reach the technical prerequisites by investing in data digitalisation and surveillance training [30].

Irrespective of the existing level of digitalisation, successful AS implementation depends on management decisions, funding and in-hospital collaboration, including continuous IT support. It further requires a strategy for implementation depending on the purpose of surveillance, and well-defined and validated protocols based on evidence and expertise that allow exchange and comparison of the surveillance data among hospitals [4,12,31,32]. Since data availability does not directly imply ‘good quality’ data, clear specifications, validation and monitoring must be part of these protocols [30,33].

Even though steps towards the development and implementation of AS are to be taken, the results of the present study indicate that widespread AS at European level will be feasible. Automated surveillance can facilitate the monitoring of all types of HAIs, with an acceptable investment in appropriate IT systems and personnel. By enhancing HAI surveillance and expanding IPC capacity to focus on interventions and preventive measures through minimising the burden of surveillance tasks, these investments could contribute to lowering the incidence of HAI across Europe, thereby reducing associated morbidity and mortality [1,6,34], and improving quality of care. Currently, AS implementation is being done centrally (i.e. data collected and algorithm application centrally) or locally (i.e. local algorithm application and sharing surveillance results centrally) [12]. An alternative solution for large scale implementation may be federated AS, where scripts for analysis are developed centrally and applied locally [32]. This strategy has the potential to decrease local surveillance burden, align AS algorithms, increase central and local oversight and improve access to local data while preserving privacy [32]. When implementing large scale AS, data interoperability is essential, which can be greatly enhanced by developments such as the European Health Data Space [35]. Promoting shared European standards, providing funding for digital infrastructure, and supporting regional training initiatives are crucial to push forward AS implementation through Europe.

Our study has some limitations. Firstly, based on the total number of European acute care hospitals given in the report of the 2022–2023 ECDC PPS (n = 8,676), the data presented here were collected from a convenience sample of 11.4% of all acute care hospitals and from 24 of 31 countries participating in the ECDC PPS, and this sample may not be representative. Secondly, due to the nature of the survey and a potentially higher interest in surveillance and/or wishful reporting of responding hospitals, the results from our analysis are more prone to overestimation, which could explain e.g. the high proportion of hospitals reporting HA-BSI surveillance. Thirdly, unfortunately, the PPS data do not provide information on how the surveillance was implemented at local level. Fourthly, 12 hospitals indicated to have a fully automated surveillance system for all seven HAIs that were part of the survey, while five of these did not have or did not report about digital data storage for parameters essential for fully AS of specific HAIs. This observation could reflect the fact that survey responders may not have understood the concept of AS, stressing the importance of training infection control staff regarding the concepts and requirements of automated HAI surveillance, in addition to on-site validation of the PPS [30]. Also, the unexpectedly high proportion of hospitals reporting fully manual surveillance and the low proportion reporting automated denominator collection may reflect limited awareness among respondents of existing automated processes (e.g. routine calculation of patient days), or differing interpretations of what constitutes automation within the PPS questionnaire. The fact that the survey may have been completed by professionals with different levels of IPC and IT expertise, may have led to varying quality of answers; additionally, the high level of incomplete questions indicates that there may have been some problems in understanding them. The direction of this bias (over or underestimation of AS degree) is unknown. Lastly, during the analysis phase, we encountered challenges interpreting the AS degree answers, which may have been related to local survey adaptations or the misinterpretation of question caused by lack of knowledge in AS. For example, it was unclear whether ‘Unknown’ was intentionally selected by the responders for AS questions on the digital and structured storage of data or represents a missing value, and which form of automation ‘Other’ refers to. Also, the response options did not allow the differentiation between deviating degrees of automation within a hospital. Even though the method that we used to calculate the levels of automation was not validated, it provides an overall impression, and the method could be used in future projects intended to measure and compare the level of AS at local, national or regional level.

## Conclusion

The results of the current study indicate that large scale adoption of AS of HAIs is feasible in European hospitals. A considerable proportion of the European hospitals having taken part in this survey, employed some form of automation. Key data elements, such as admission dates, discharge dates and microbiology results were digitally stored by most hospitals. To advance AS operationalisation in Europe, increasing knowledge on the concepts of AS and requirements for implementation, and developing tailored strategies depending on the level of digitalisation, type and size of hospital, and the hospitals' organisational needs will be important. Additionally, well-defined and validated protocols for AS implementation are needed, as well as data standardisation and interoperability across Europe. The future of HAI surveillance in Europe relies on cross-country collaboration and sustainable investment in digital infrastructure, standardised data systems, and training infection prevention teams to use automated tools.

## Data Availability

Data available upon request: Aggregated data are available through a data access request to ECDC. Third-party access to case-based EU/EEA surveillance data is currently under legal review to ensure alignment with Regulation (EU) 2022/2371 on serious cross-border threats to health and is hence suspended. Therefore, no new requests for case-based data will be processed until further notice.

## ECDC PPS study group on automated surveillance of HAIs

Seven Johannes Sam Aghdassi: Charité – Universitätsmedizin Berlin, corporate member of Freie Universität Berlin and Humboldt-Universität zu Berlin, Institute of Hygiene and Environmental Medicine, Berlin, Germany; National Reference Centre for Surveillance of Nosocomial Infections, Berlin, Germany. Lucie Bareková: National reference centre for healthcare associated infections, Prague, Czech Republic. Zrinka Bošnjak: University Hospital Centre Zagreb, Croatia. Mireia Cantero: Preventive Medicine Department, Hospital Universitario Puerta de Hierro-Majadahonda, Madrid, Spain. Elina Dobreva: National Center of Infectious and Parasitic Diseases (NCIPD), Sofia, Bulgaria; National Reference Laboratory of Control and Monitoring of Antibiotic Resistance (NRL-CMAR). Ólafur Guðlaugsson: Landspitali University Hospital Reykjavik, Iceland. Irena Klavs: National Institute of Public Health, Ljubljana, Slovenia. Katrien Latour: Sciensano, Brussels, Belgium. Slavka Litvová: Regionálny úrad verejného zdravotníctva so sídlom v Trenčíne, Trenčín, Slovakia. Stephen Murchan: HSE Health Protection Surveillance Centre, Dublin, Ireland. Alexandre Mzabi: Directorate of Health, Ministry of Health and Social Security, Luxembourg. Dulce Pascoalinho: Intensive Medicine Department, Unidade Local de Ssaúde do Litoral Alentejano, Santiago do Cacém, Portugal; Infection Prevention and Control and Antimicrobial Stewardship Unit, Unidade Local de Ssaúde do Litoral Alentejano, Santiago do Cacém, Portugal. Radica Raičević: Institut za javno zdravlje Crne Gore, Podgorica, Montenegro. Elizabeth Anne Scicluna: Mater Dei Hospital, Msida, Malta.

## ^‡^Note

This designation is without prejudice to positions on status, and is in line with UNSCR 1244/99 and the IСJ Opinion on the Kosovo declaration of independence.

## Supplementary Data

6249000 Supplement
