# Supplementary material for: Current state and potential of hospitals for automated healthcare-associated infection surveillance: data from 24 European countries, 2022 to 2023
Source: Euro Surveill. 2026 May 14;31(19):2500736. doi: 10.2807/1560-7917.ES.2026.31.19.2500736 (PMC13179479; doi:10.2807/1560-7917.ES.2026.31.19.2500736)
Supplement: Supplement [file 25-00736_VAN_ROODEN_Supplement.pdf]

This supplementary material is hosted by *Eurosurveillance* as supporting information alongside the article "*The current state and promise of hospitals throughout Europe for automated healthcare-associated infection surveillance"*, on behalf of the authors, who remain responsible for the accuracy and appropriateness of the content.

The same standards for ethics, copyright, attributions and permissions as for the article apply. Supplements are not edited by *Eurosurveillance* and the journal is not responsible for the maintenance of any links or email addresses provided therein.

Supplementary Material 1: Questions on automated surveillance of healthcare-associated infections and definitions of the response options as indicated in the protocol from the 2022/2023 European Centers of Disease Prevention and Control point prevalence survey of healthcare-associated infections and antimicrobial use in European acute care hospitals

1) Current degree of automation of surveillance of HAIs.

Indicate for each listed type of HAI to what degree the surveillance of this type of HAI is automated in your hospital:

0. **Fully manual:** Fully manual surveillance, selection of patients that should be included in the surveillance (e.g. based on device use or procedures) and detection of HAI is performed by manually reviewing charts.
1. ***Automated denominator collection:*** Automated rule-based routine selection of procedures or patient- days to be included in the surveillance, e.g. based on admission to specific wards, surgical procedures or use of devices such as central lines; Codes are selected without manual steps and directly linked to a digital record for surveillance purposes. Subsequently charts are manually reviewed to detect HAI in the selected patients.
2. ***Semi-automated:*** Automated selection of patients in surveillance (as in #1) AND an automated algorithm flags patients with a high probability of an HAI that require manual confirmation of HAI presence, based on information extracted from electronic health records and linked to a digital record for surveillance purposes.
3. ***Fully automated:*** Automated selection of patients in surveillance (as in #1) AND fully automated algorithm for detection of HAI based on information extracted from electronic health records. This means that no manual selection or confirmation step is necessary.
4. ***Other:*** Electronically available databases are used to either preselect patients to be included in the surveillance (denominator collection) and/or preselect patients that require manual confirmation of HAI presence (e.g. from microbiology database) without automated direct linkage to an electronic surveillance record (still requiring manual steps for the selection process).
9. ***Not performed***: Surveillance is not performed for this type of HAI.

Note that the term `automation’ in this question refers to the process of case finding of HAIs and denominator selection, not the electronic data linkage to case-mix variables (e.g. age, ASA score, wound class in SSI surveillance) to automate the surveillance.

The current degree of automation of surveillance is asked for following types of HAI:

Surgical site infection (SSI): surveillance of SSIs for one or more operation categories.

Healthcare-associated bloodstream infection (HA-BSI): surveillance of BSIs with onset on day 3 of the hospital stay or 48 hours or more after admission, with or without determination of the origin (source) of the BSI

Central line-associated bloodstream infection (CLABSI) (synonym: central vascular catheter (CVC)-associated bloodstream infection) or CVC-related bloodstream infection: CVC/central line-*associated* refers to the presence of a CVC within 48 hours before onset of a primary BSI (i.e. a BSI which is not secondary to another infection site); CVC-*related* bloodstream infection refers to microbiological or clinical criteria to determine the origin of the bloodstream infection (see definition of BSI origin and case definition of CRI3).

Catheter-associated urinary tract infections (CAUTI): urinary tract infections with presence of a urinary catheter within 7 days before onset of the urinary tract infection

Healthcare-associated pneumonia (HA-PN): pneumonia (see case definition) with onset on day 3 of the hospital stay or 48 hours or more after admission, with or without determination of an association to invasive device use.

Ventilator-associated pneumonia (VAP), or intubation-associated pneumonia (IAP), see case definitions.

Clostridoides difficile infections (CDI)

2) Feasibility of automated HAI surveillance.

Feasibility of the data collection for automated HAI surveillance. Automated surveillance requires extraction of data from electronic health records in a structured – and preferably interoperable – format. For the data sources listed below, please indicate:

Are the data stored digitally: the data exist in a digital subsystem. YH=Yes, hospital-wide; YW=Yes, in specific wards only; N=No; UNK=Unknown

If yes: indicate whether the data are stored in a structured format (e.g., not as free text notes but as coded or standardised information): Y=Yes / N=No / NA=Not applicable / UNK=Unknown. Examples of structured and well-defined data include date field in standard format (YYYY-MM-DD), ICD-10 diagnosis codes, ATC codes to specify prescribed medication; Example of data not structured is reporting of extraction of catheter in free text fields only.

List of data sources:

Surgical procedures: procedure code such as ICD-10, date of surgery

Admission and discharge dates, hospital level

Admission and discharge dates, unit level

Use of central lines: date of insertion and removal, type*

Use of mechanical ventilation or intubation: start date, end date

Use of urinary catheters: date of insertion/removal*

Microbiology culture results (culture result, date of sampling, specimen type)

Antimicrobial prescriptions: antimicrobial name or code (preferrable ATC code level 5), start date, end date

* For central lines and urinary catheters, if only the insertion date but not the removal date is known, please indicate ‘Yes’.

3) Excerpt of the automated surveillance questions from the technical document of the 2022/2023 ECDC point prevalence survey

Current degree of automation of surveillance of HAIs:

	0.Fullymanual	1.Automated denominator	2.Semi - automated	3.Fully automated	4. other	9. Not performed
Surgical site infection*						
Healthcare -associated BSI						
Central line -associated BSI						
Catheter -associated UTI						
Healthcare -associated pneumonia						
Ventilator -associated pneumonia						
Clostridoides difficile infection						

Feasibility of automated HAI surveillance:

Data source	Data are stored digitally (a)	Data are structured and well -defined (b)
Surgical procedures (procedure code such as ICD -10, date of surgery)		
Admission and discharge dates, hospital level		
Admission and discharge dates, unit level		
Use of central lines (date of insertion/extraction, type)		
Use of mechanical ventilation (start date, end date)		
Use of urinary catheters* (date of insertion/extraction)		
Microbiology culture results (culture result, date, specimen type)		
Antimicrobial prescriptions (ATC code, start date, end date)		

https://www.ecdc.europa.eu/sites/default/files/documents/antimicrobial-use-healthcare-associated-infections-point-prevalence-survey-version6-1.pdf (page 11)

Supplementary Material 2: Calculation of summarising levels of automated surveillance of healthcare-associated infections and digital storage of key data elements for automation.

1) Current level of automation of HAI surveillance per hospital:

The current degree of automated surveillance (AS) of healthcare-associated infections (HAI) was assessed through seven AS questions, all using the same response options (see below). For the calculation of an overall level of AS, the lowest value was assigned to the category *Unknown*, because this represents missing values. The category *Not performed* was assigned a score of one, while a score of two was allocated to both *Other* and *Fully manual*, because *Other* indicated that surveillance was conducted to some extent (which represents a higher level of activity compared to *Unknown* or *Not Performed*), but without being applicable to one of the other response options. To account for this uncertainty and to adopt a conservative approach, we assumed the worst scenario for *Other* — that surveillance is conducted manually and only in parts of the hospital. To reflect this cautious interpretation, the value for *Other* was aligned with *Fully manual*. Additionally, the categories *Automated denominator*, *Semi-automated* and *Fully automated* were assigned a value of three, four and five, respectively.

1) Considered questions (n=7):
    - A) Current degree of automation of surveillance of HA-BSI
    - B) Current degree of automation of surveillance of CAUTI
    - C) Current degree of automation of surveillance of CDI
    - D) Current degree of automation of surveillance of CLABSI
    - E) Current degree of automation of surveillance of HAP
    - F) Current degree of automation of surveillance of SSI
    - G) Current degree of automation of surveillance of VAP
2) Possible responses and assigned values
    - No response (*unknown*) → 0
    - Not performed → 1
    - Other → 2
    - Fully manual → 2
    - Automated denominator → 3
    - Semi-automated → 4
    - Fully automated → 5
3) Calculation of normalised levels (0-100):
    - Add the values of the responses to questions A + B + C + D + E + F + G
    - Calculate the percentage of the achieved score relative to the maximum possible score of 35 (7 questions * 5 points) and multiply by 100 to express the value on a scale from 0 to 100.

2) Current level of digital storage of key data elements for automation of HAI surveillance per hospital:

The feasibility of automated HAI surveillance was assessed through eight AS questions related to the digitisation of patient records, all using the same response options (see below). In the questionnaire, if hospitals answered *Yes* to the initial question, *"Is the data stored digitally?"*, they should then respond to the follow-up question, *"Is the data structured and well-defined?"*. Since only a subset of hospitals provided responses to the follow-up questions, these follow-up data were excluded from the calculation of an overall feasibility level for automated HAI surveillance. This approach ensured consistency and comparability across all hospitals when determining feasibility. For the calculation of

an overall level of feasibility based on the questions regarding the storage of data, the lowest value was assigned to the category *Unknown*, as this could mean both missing values and an absence of knowledge regarding the digital storage of data. Here (in contrast to the questions on the current degree of AS), *Unknown* was indeed a response option, but it could not be differentiated from missing values that were also stored as *Unknown* in the dataset. Subsequently, the categories *No*, *Yes, in specific wards* and *Yes, hospital-wide* were assigned a value of one, two and three, respectively.

1) Considered questions (n=8):
   - A) Data on admission and discharge dates are stored digitally at hospital level
   - B) Data on admission and discharge dates are stored digitally at ward level
   - C) Data on antimicrobial prescriptions (ATC code, start date, end date) are stored digitally
   - D) Data on the use of central lines (date of insertion/extraction and CVC type) are stored digitally
   - E) Data on microbiology (culture results, date of sampling, specimen type) are stored digitally
   - F) Data on the use of mechanical ventilation (start and end dates) are stored digitally
   - G) Data on surgical procedures (procedure code such as ICD-10, date of surgery) are stored digitally
   - H) Data on the use of urinary catheters (date of insertion/extraction) are stored digitally
2) Possible responses and assigned values
   - Unknown or no response (*unknown*) → 0
   - No → 1
   - Yes, in specific wards → 2
   - Yes, hospital-wide → 3
3) Calculation of normalised levels (0-100):
   - Add the values of the responses to questions A + B + C + D + E + F + G + H
   - Calculate the percentage of the achieved score relative to the maximum possible score of 24 (8 questions * 3 points) and multiply by 100 to express the value on a scale from 0 to 100.

Table S1: Descriptive data of included European acute care hospitals stratified by geographic regions. Europe, 2022-2023, n=992.

	East (n=318)		North (n=124)		South (n=407)		West (n=143)		p-value
**Hospital ownership, n (%)**									
Public	269	84.6	105	84.7	325	79.9	60	42.0	<0.001
Private for-profit	22	6.9	9	7.3	72	17.6	18	12.6	
Private not-for-profit	10	3.1	9	7.3	6	1.5	59	41.3	
Other/unknown	17	5.3	1	0.8	4	1.0	6	4.2	
**Hospital type, n (%)**									
Primary level	88	27.7	28	22.6	58	14.3	76	53.1	<0.001
Secondary level	104	32.7	46	37.1	198	48.6	35	24.5	
Tertiary level	70	22.0	33	26.6	111	27.3	12	8.4	
Specialised	55	17.3	17	13.7	39	9.6	20	14.0	
Unknown	1	0.3	0	0	1	0.2	0	0	
**No. hospital beds, median [IQR],** 100% response	404	249-663	171	87-307	214	112-420	240	128-415	<0.001
**No. ICU beds, median [IQR],** 97.6-99.3% response	14	6-32	6	0-12	11	5-22	9	5-21	<0.001
**No. patient days/year, median [IQR],** 87.1-100% response	79139	47703-136333	48561	21249-9292	43245	21249-93928	53239	26245-90213	<0.001

* chi-square test (for categorical variables) and Kruskal-Wallis test and post-hoc Dunn-Bonferroni test (for continuous variables). Abbreviations: ICU - intensive care unit; IQR - interquartile range

Table S2: Levels of automated surveillance of healthcare-associated infections and digital storage of key data elements in European acute care hospitals, stratified by geographic regions and hospital characteristics. Europe, 2022-2023, n=992.

	Levels of automated surveillance and digital data storage (median and IQR)			
**Geographic region**	Level of AS	**p-value***	Level of digital data storage	**p-value***
East (n=318)	40.0 (40.0-51.4)	0.012*[1]	70.8 (50.0-91.7)	0.021*[2]
North (n=124)	40.0 (34.3-57.1)		70.8 (58.3-95.8)	
South (n=407)	40.0 (31.4-42.9)		75.0 (58.3-95.8)	
West (n=143)	40.0 (31.4-57.1)		70.8 (50.0-87.5)	
**Hospital ownership**				
Public (n=759)	40.0 (34.3-48.6)	0.007*[3]	75.0 (58.3-91.7)	0.445
Private for-profit (n=121)	40.0 (28.6-41.4)		75.0 (62.5-97.9)	
Private not-for-profit (n=84)	40.0 (32.1-62.9)		75.0 (50.0-91.7)	
Other/unknown (n=27)	37.1 (28.6-42.9)		70.8 (50.0-87.5)	
**Hospital type**				
Primary (n=250)	40.0 (31.4-42.9)	<0.001*[4]	70.8 (58.3-91.7)	<0.001*[5]
Secondary (n=383)	40.0 (31.4-45.7)		75.0 (50.0-91.7)	
Tertiary (n=226)	40.0 (40.0-60.0)		85.4 (69.8-100)	
Specialised (n=131)	40.0 (34.3-42.9)		66.7 (50.0-87.5)	
**Hospital size**				
<= 250 beds (n=466)	40.0 (31.4-45.7)	<0.001*[6]	75.0 (58.3-91.7)	0.662
250-499 beds (n=282)	40.0 (37.1-52.1)		75.0 (57.3-92.7)	
500-999 beds (n=184)	40.0 (40.0-57.1)		75.0 (58.3-95.8)	
>= 1000 beds (n=60)	40.0 (40.0-53.6)		75.0 (46.9-91.7)	

Legend: *Kruskal-Wallis test and post-hoc Dunn-Bonferroni test (significant results of 1: east vs. south (p=0.011), 2: no significant pairs, 3: private for-profit vs. public (p=0.035), private for-profit vs. private not-for-profit (p=0.043), 4: tertiary vs. primary (p<0.001), tertiary vs. secondary (p<0.001), tertiary vs. specialised (p=0.001), 5: tertiary vs. primary (p<0.001), tertiary vs. secondary (p<0.001), tertiary vs. specialised (p<0.001), 6: <=250 beds vs. 250-499 beds (p=0.003), <=250 beds vs.500-999 beds (p=0.003), <=250 beds vs. >=1000beds (p=0.029)).

Table S3: Comparison of hospitals with complete response to automated surveillance questions with hospitals with at least one missing or unknown response. Europe, 2022-2023, n=992

	Complete response (n=680)		>=1 missing / unknown (n=312)		Complete response rate*
**Geographic region, n (%)**					
East	228	33.5	90	28.8	71.7
North	83	12.2	41	13.1	66.9
South	312	45.9	95	30.4	76.7
West	57	8.4	86	27.6	39.9
**Hospital ownership, n (%)**					
Public	533	78.5	226	72.4	70.2
Private for-profit	88	13.0	33	10.6	72.7
Private not-for-profit	39	5.7	45	14.4	46.4
Other/unknown	19	2.8	8	2.6	70.4
**Hospital type, n (%)**					
Primary level	150	22.1	100	32.1	60.0
Secondary level	267	39.3	116	37.2	69.7
Tertiary level	157	23.1	69	22.1	70.4
Specialised	105	15.4	26	8.3	80.2
Unknown	1	0.1	1	0.3	50.0
**No. hospital beds, median [IQR],** 100% response	260	128-492	292	148-494	NA
**No. patient days/year, median [IQR],** 96.8-99.4% response	58359	26080-103363	62343	31898-117411	NA

Legend: *Complete response rate = number of hospitals with complete response / number of responding hospitals x 100.
Abbreviations: IQR – interquartile range; NA – not applicable

Table S4: Number of hospitals with missing or unknown responses per question on automated surveillance. Europe, 2022-2023, n=312.

**Degree of AS**		
**Geographic region, n (%)**		
East	10	3.2
North	3	1.0
South	9	2.9
West	6	2.9
**Healthcare-associated infection, n (%)**		
HA-BSI	8	2.6
CAUTI	9	2.9
CDI	10	3.2
CLABSI	10	3.2
HAP	15	4.8
SSI	10	3.2
VAP	16	5.1
**Digital data storage**		
**Geographic region, n (%)**		
East	62	19.9
North	26	8.3
South	52	16.7
West	66	21.2
**Key data elements, n (%)**		
Admission and discharge dates (hospital-level)	77	24.7
Admission and discharge dates (ward-level)	78	25.0
Antimicrobial prescriptions	132	42.3
Use of central lines	86	27.6
Microbiology results	77	24.7
Use of mechanical ventilation	114	36.5
Surgical procedures	93	29.8
Use of urinary catheters	92	29.5
**Structured data storage**		
**Geographic region, n (%)**		
East	72	23.1
North	34	10.9
South	77	24.7
West	84	26.9
**Key data elements, n (%)[#]**		
Admission and discharge dates (hospital-level), n=226[*]	104	46.0
Admission and discharge dates (ward-level), n=223[*]	100	44.8
Antimicrobial prescriptions, n=131[*]	79	60.3

Use of central lines, n=135*	80	59.3
Microbiology results, n=218*	98	45.0
Use of mechanical ventilation, n=123*	72	58.5
Surgical procedures, n=183	94	51.4
Use of urinary catheters, n=126	73	57.9

Legend: #Here, the denominator is the number of hospitals, which responded with *yes* to the related question on the digital storage of key data elements for AS, because the follow-up question on the structured storage of data applied only to those hospitals. *The number of hospitals, which responded with yes to the related question on the digital storage of key data elements for AS. Abbreviations: AS: automated surveillance; CAUTI: catheter-associated urinary tract infection; CDI: *Clostridioides difficile* infection; CLABSI: central line-associated bloodstream infection; HA-BSI: healthcare-associated bloodstream infection; HAP: healthcare-associated pneumonia; SSI: surgical site infection; VAP: ventilator-associated pneumonia

Table S5: Responses to automated surveillance questions from hospitals with complete response and all responding hospitals. Europe, 2022-2023, n=992

		Complete response, n (% of 680 hospitals)		Total, n (% of 990 hospitals)		p- value*
**Current degree of AS**						
**HA-BSI**						
	Unknown	0	0	8	-	
	Not performed	110	16.2	155	15.8	0.89
	Other	83	12.2	105	10.7	
	Fully manual	297	43.7	433	44.1	
	Automated denom.	70	10.3	100	10.2	
	Semi-automated	107	15.7	168	17.1	
	Fully automated	13	1.9	23	2.3	
**CAUTI**						
	Unknown	0	0	9	-	
	Not performed	162	23.8	245	25.0	0.814
	Other	76	11.2	92	9.4	
	Fully manual	297	43.7	419	42.7	
	Automated denom.	40	5.9	60	6.1	
	Semi-automated	92	13.5	147	15.0	
	Fully automated	13	1.9	20	2.0	
**CDI**						
	Unknown	0	0	10	-	
	Not performed	93	13.7	131	13.4	0.862
	Other	85	12.5	105	10.7	
	Fully manual	302	44.4	450	45.9	
	Automated denom.	81	11.9	112	11.4	
	Semi-automated	102	15.0	155	15.8	
	Fully automated	17	2.5	29	3.0	
**CLABSI**						
	Unknown	0	0	10	-	
	Not performed	118	17.4	162	16.5	0.830
	Other	83	12.2	104	10.6	
	Fully manual	309	45.4	448	45.7	
	Automated denom.	60	8.8	89	9.1	
	Semi-automated	96	14.1	155	15.8	
	Fully automated	14	2.1	24	2.4	
**HAP**						
	Unknown	0	0	15	-	
	Not performed	219	32.2	314	32.2	0.881
	Other	69	10.1	86	8.8	
	Fully manual	271	39.9	385	39.5	

	Automated denom.	36	5.3	53	5.4	
	Semi-automated	75	11.0	121	12.4	
	Fully automated	10	1.5	18	1.8	
**SSI**						
	Unknown	0	0	10	-	
	Not performed	155	22.8	210	21.4	0.582
	Other	71	10.4	87	8.9	
	Fully manual	280	41.2	422	43.1	
	Automated denom.	68	10.0	88	9.0	
	Semi-automated	97	14.3	156	15.9	
	Fully automated	9	1.3	19	1.9	
**VAP**						
	Unknown	0	0	16	-	
	Not performed	204	30.0	285	29.3	0.788
	Other	71	10.4	89	9.1	
	Fully manual	271	39.9	391	40.1	
	Automated denom.	41	6.0	57	5.9	
	Semi-automated	84	12.4	135	13.9	
	Fully automated	9	1.3	19	2.0	

* chi-square test (for categorical variables). Hospitals responding with ‘unknown’ were excluded from the calculation of proportions to enable comparability between cohorts. Abbreviations: AS – automated surveillance; CAUTI - catheter-associated urinary tract infection; CDI - Clostridioides difficile infection; CLABSI - central line-associated bloodstream infection; HA-BSI - healthcare-associated bloodstream infection; HAP - healthcare-associated pneumonia; SSI - surgical site infection; VAP - ventilator-associated pneumonia

Figure S1: Reported degree of automated surveillance of different healthcare-associated infections in 992 European acute care hospitals, stratified by geographic regions

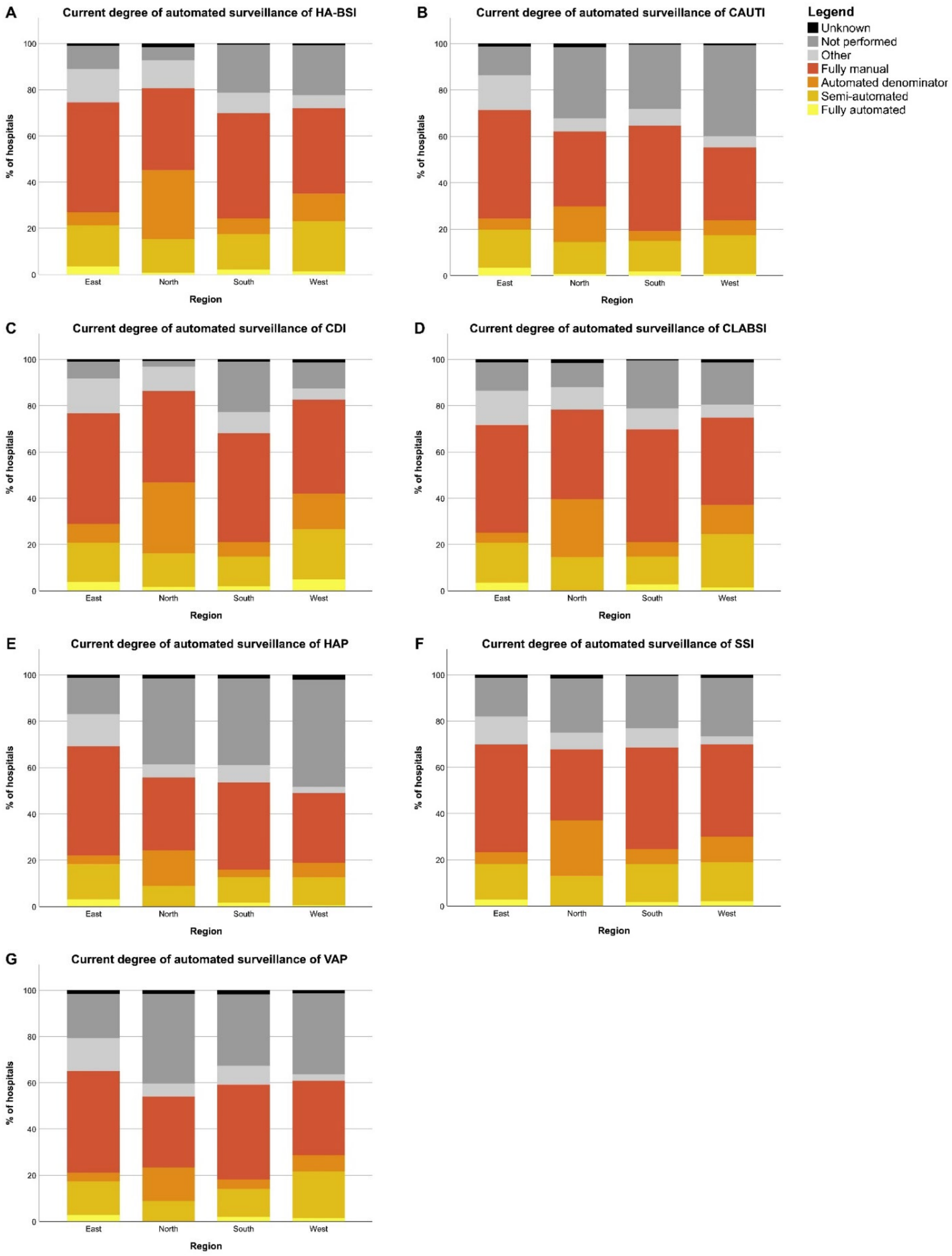

A: healthcare-associated bloodstream infection (HA-BSI), B: catheter-associated urinary tract infection (CAUTI), C: *Clostridioides difficile* infection (CDI), D: central line-associated bloodstream infection (CLABSI), E: healthcare-associated pneumonia (HAP), F: surgical site infection (SSI), G: ventilator-associated pneumonia (VAP); Footnote: No hospitals per region: n=318 (east), n=124 (north), n=407 (south), n=143 (west).

Figure S2: Reported digital storage of different key data elements for automated surveillance of healthcare-associated infections in 992 European acute care hospitals, stratified by geographic regions

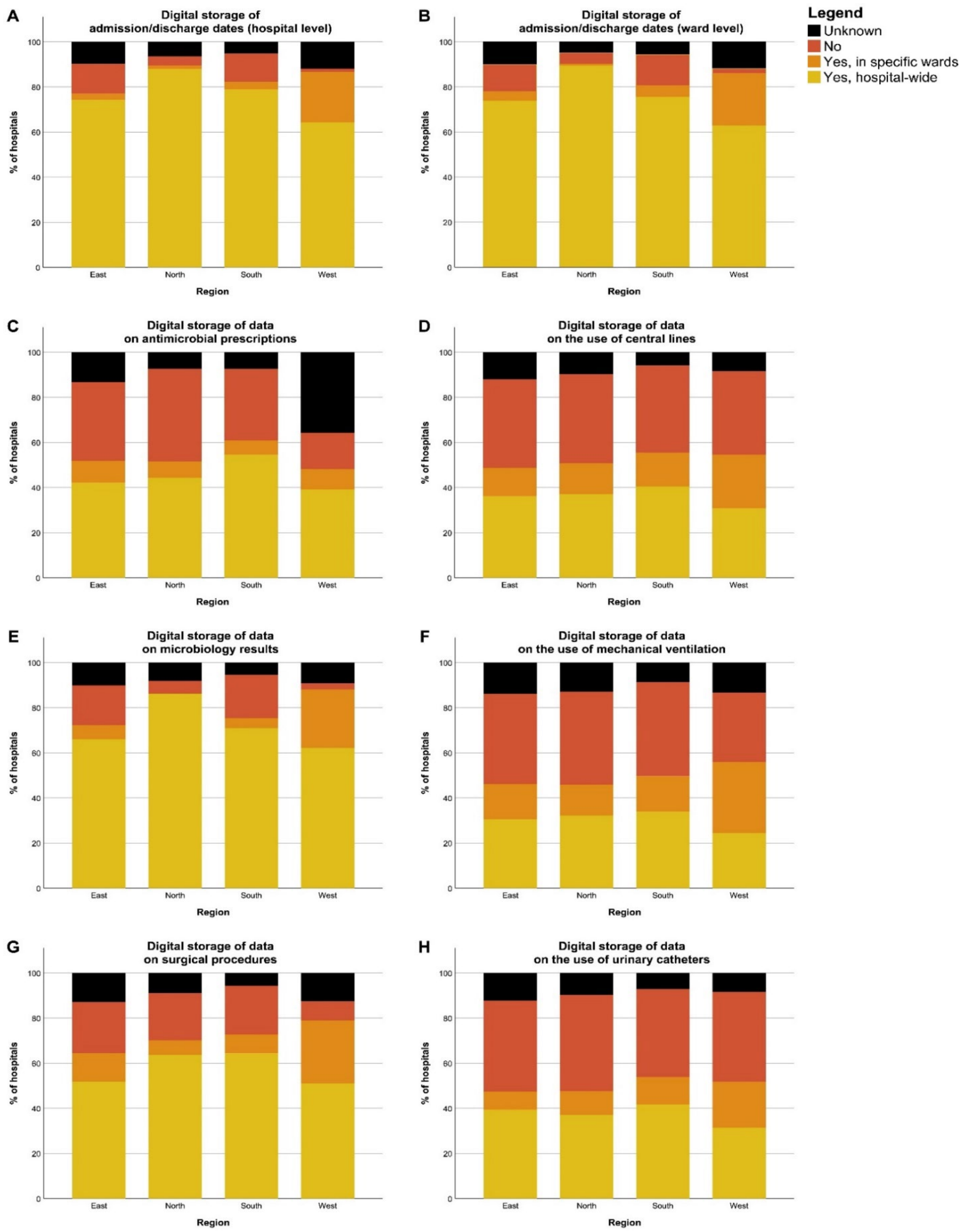

A: admission/discharge dates (hospital level), B: admission/discharge dates (ward level), C: antimicrobial prescriptions, D: central lines, E: microbiology results, F: mechanical ventilation, G: surgical procedures, H: urinary catheters. Footnote: No. hospitals per region: n=318 (east), n=124 (north), n=407 (south), n=143 (west). Hospitals answering with “No” to the question “Is the data stored digitally?” (Figure S2) could respond with “NA” (=not applicable) to the follow-up question “Is the data stored in a structured and well-defined format?” (Figure S3).

Figure S3: Reported structured digital storage of different key data elements for automated surveillance of healthcare-associated infections in 992 European acute care hospitals, stratified by geographic regions

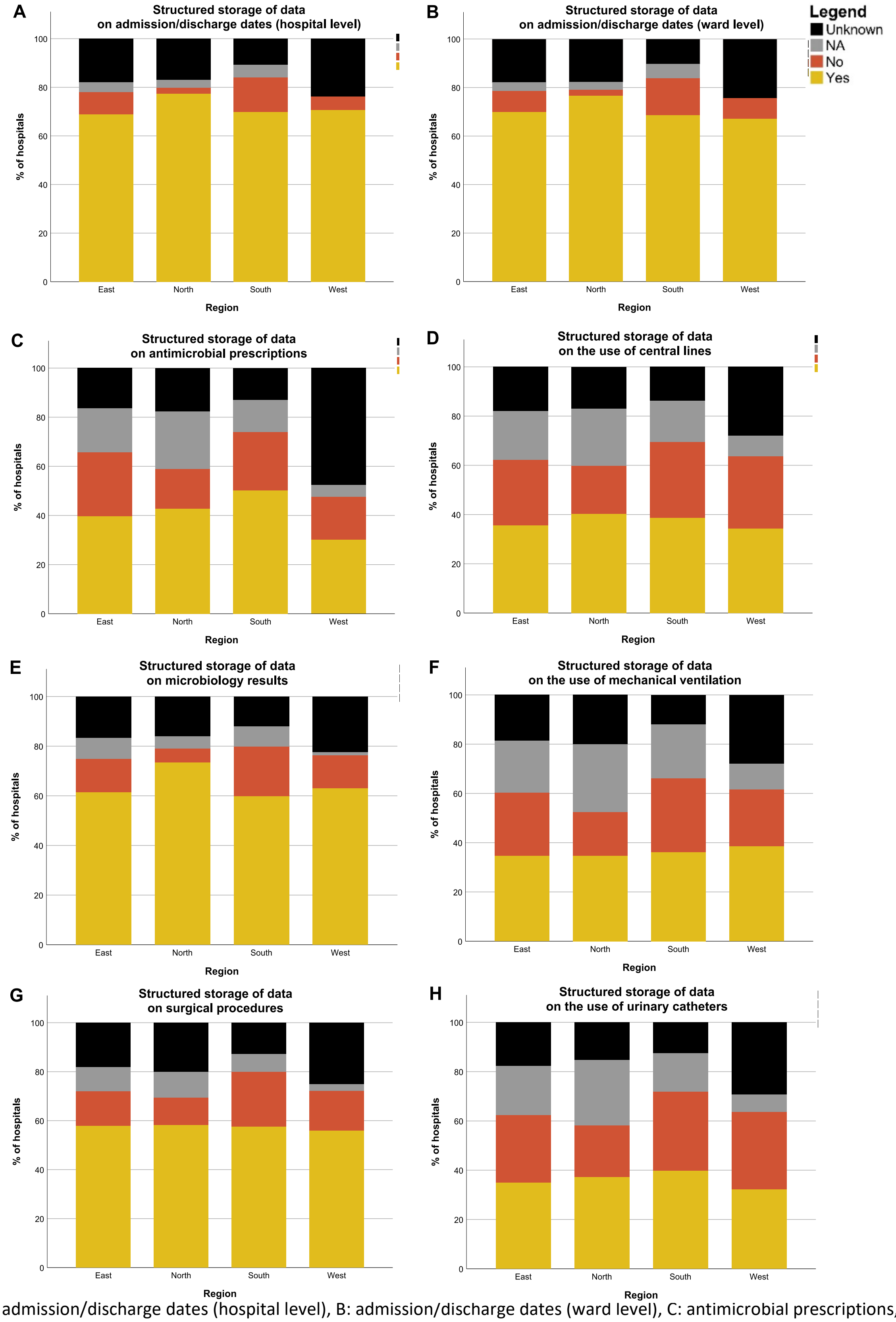

A: admission/discharge dates (hospital level), B: admission/discharge dates (ward level), C: antimicrobial prescriptions, D: central lines, E: microbiology results, F: mechanical ventilation, G: surgical procedures, H: urinary catheters. Footnote: No. hospitals per region: n=318 (east), n=124 (north), n=407 (south), n=143 (west). Hospitals answering with “No” to the question “Is the data stored digitally?” (Figure S2) could respond with “NA” (=not applicable) to the follow-up question “Is the data stored in a structured and well-defined format?” (Figure S3).

Fig. S4: Reported digital storage of different key data elements for automated surveillance of healthcare-associated infections in 992 European acute care hospitals, stratified by the reported degree of automation

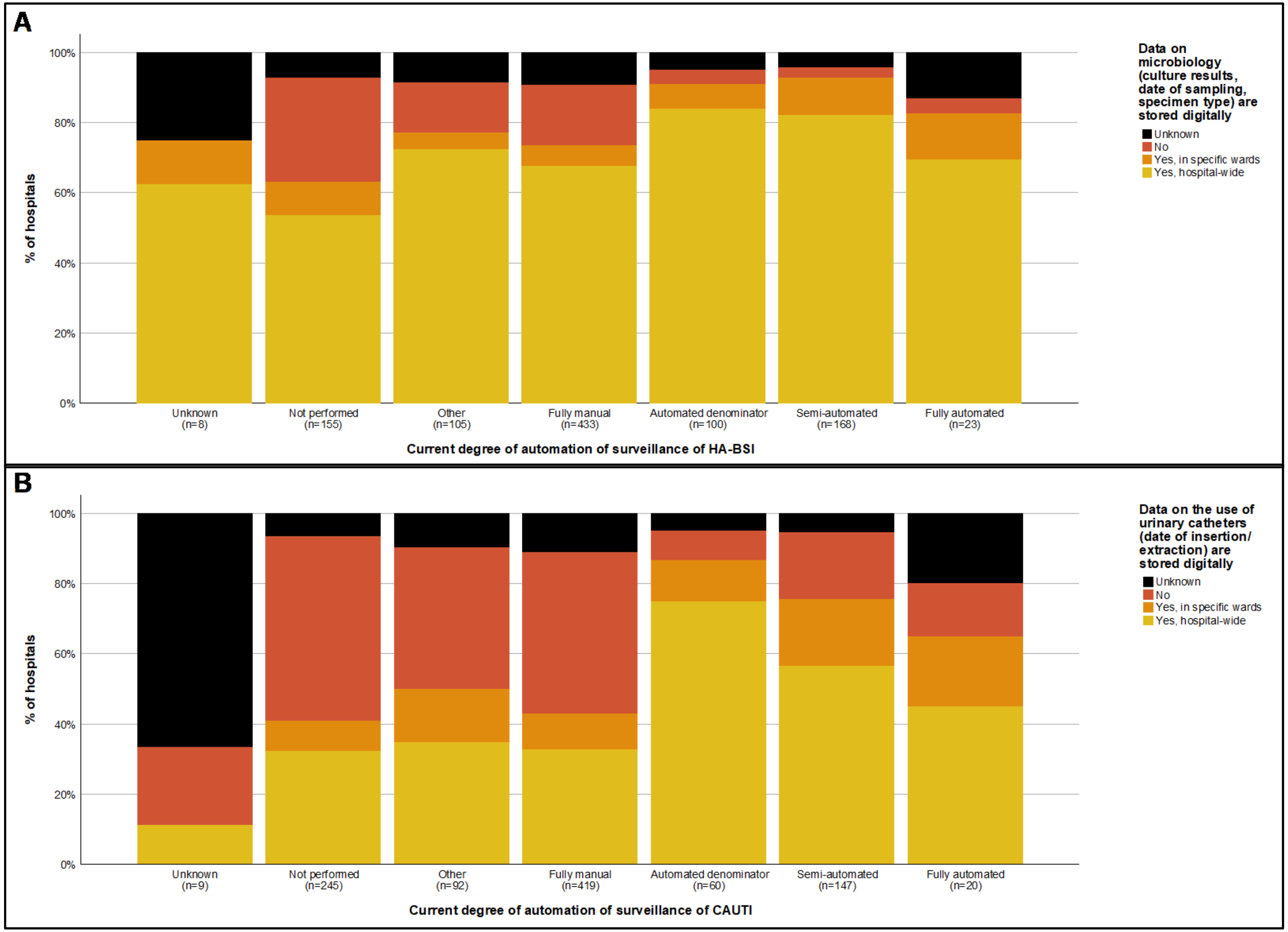

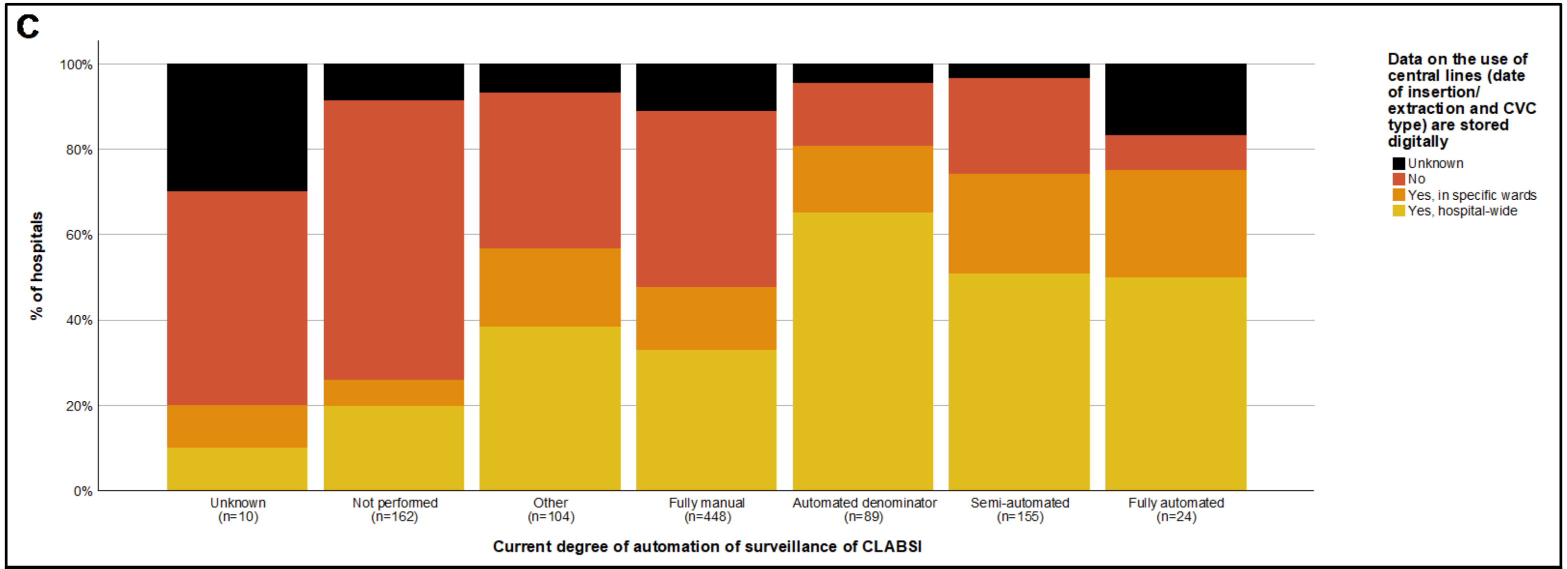
C
100%
80%
60%
40%
20%
0%
% of hospitals
Unknown (n=10)
Not performed (n=162)
Other (n=104)
Fully manual (n=448)
Automated denominator (n=89)
Semi-automated (n=155)
Fully automated (n=24)
Current degree of automation of surveillance of CLABSI
Data on the use of central lines (date of insertion/ extraction and CVC type) are stored digitally
Unknown
No
Yes, in specific wards
Yes, hospital-wide

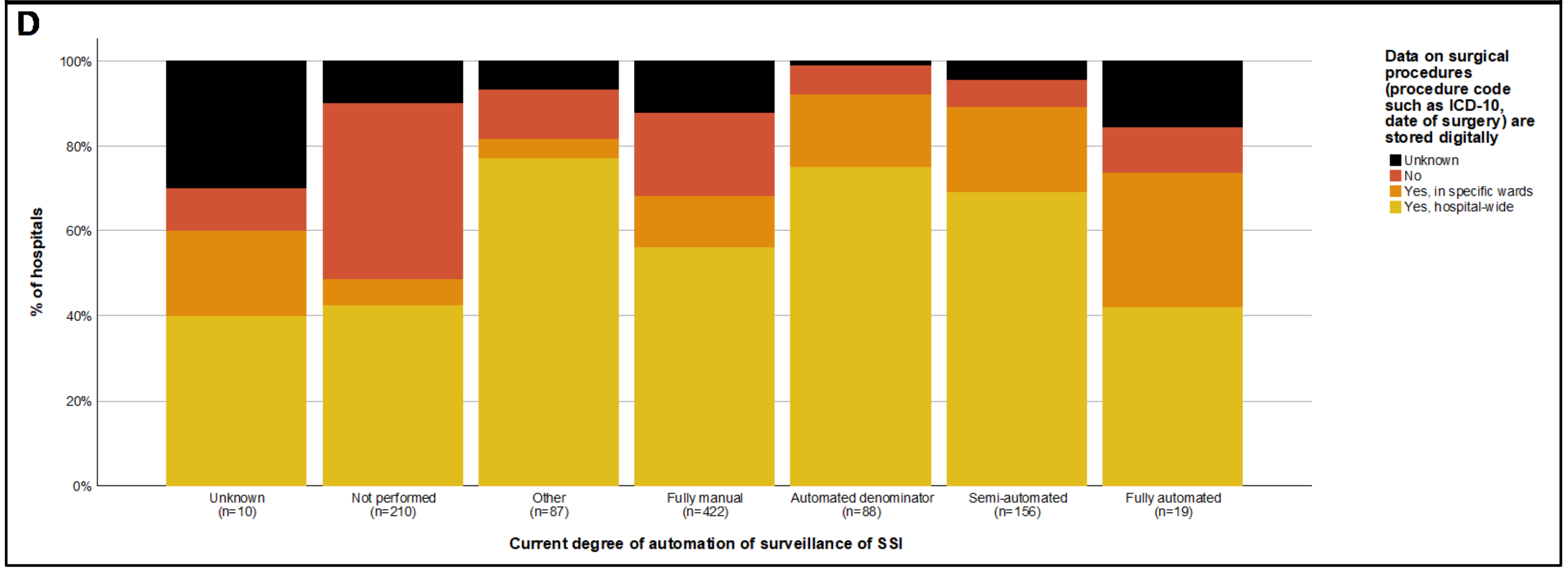
D
100%
80%
60%
40%
20%
0%
% of hospitals
Unknown (n=10)
Not performed (n=210)
Other (n=87)
Fully manual (n=422)
Automated denominator (n=88)
Semi-automated (n=156)
Fully automated (n=19)
Current degree of automation of surveillance of SSI
Data on surgical procedures (procedure code such as ICD-10, date of surgery) are stored digitally
Unknown
No
Yes, in specific wards
Yes, hospital-wide

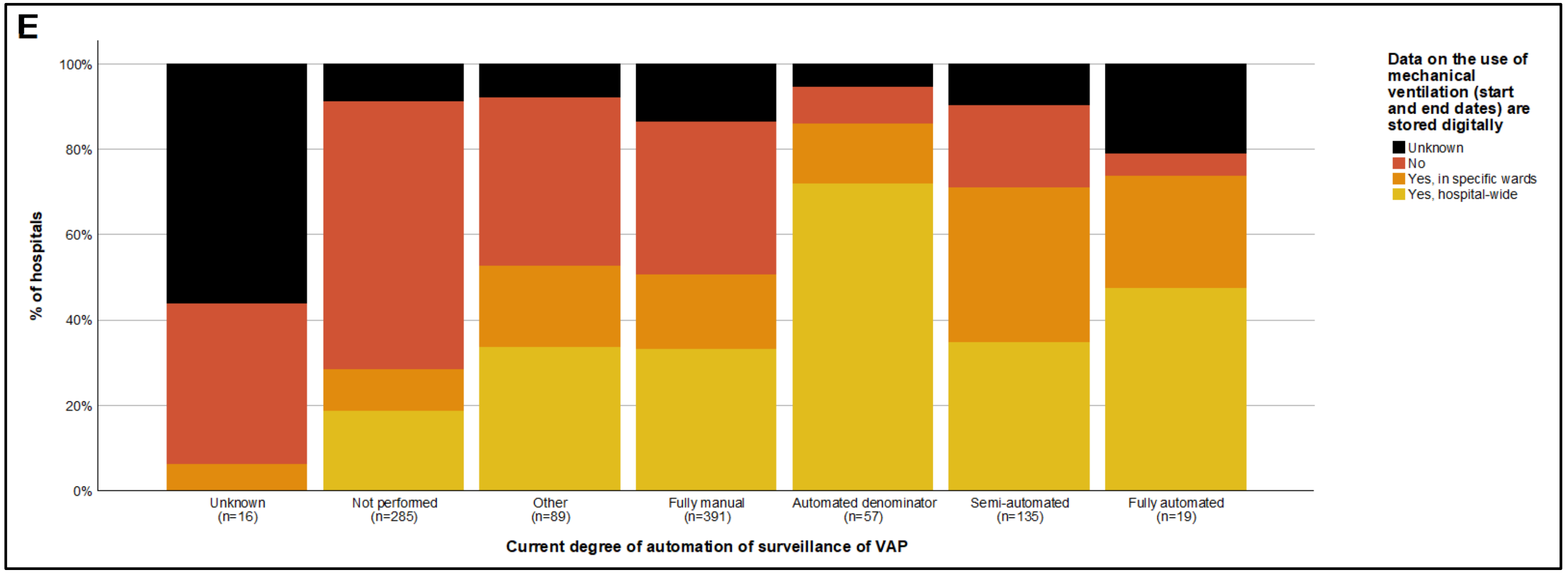

A: healthcare-associated bloodstream infection (HA-BSI), B: catheter-associated urinary tract infection (CAUTI), C: central line-associated bloodstream infection (CLABSI), D: surgical site infection (SSI), E: ventilator-associated pneumonia (VAP); Footnote: Number of hospitals are presented below the bars.

Figure S5: Reported digital storage of key data elements for automated surveillance of healthcare-associated infections in 992 European acute care hospitals, stratified by degree of automation and geographic regions

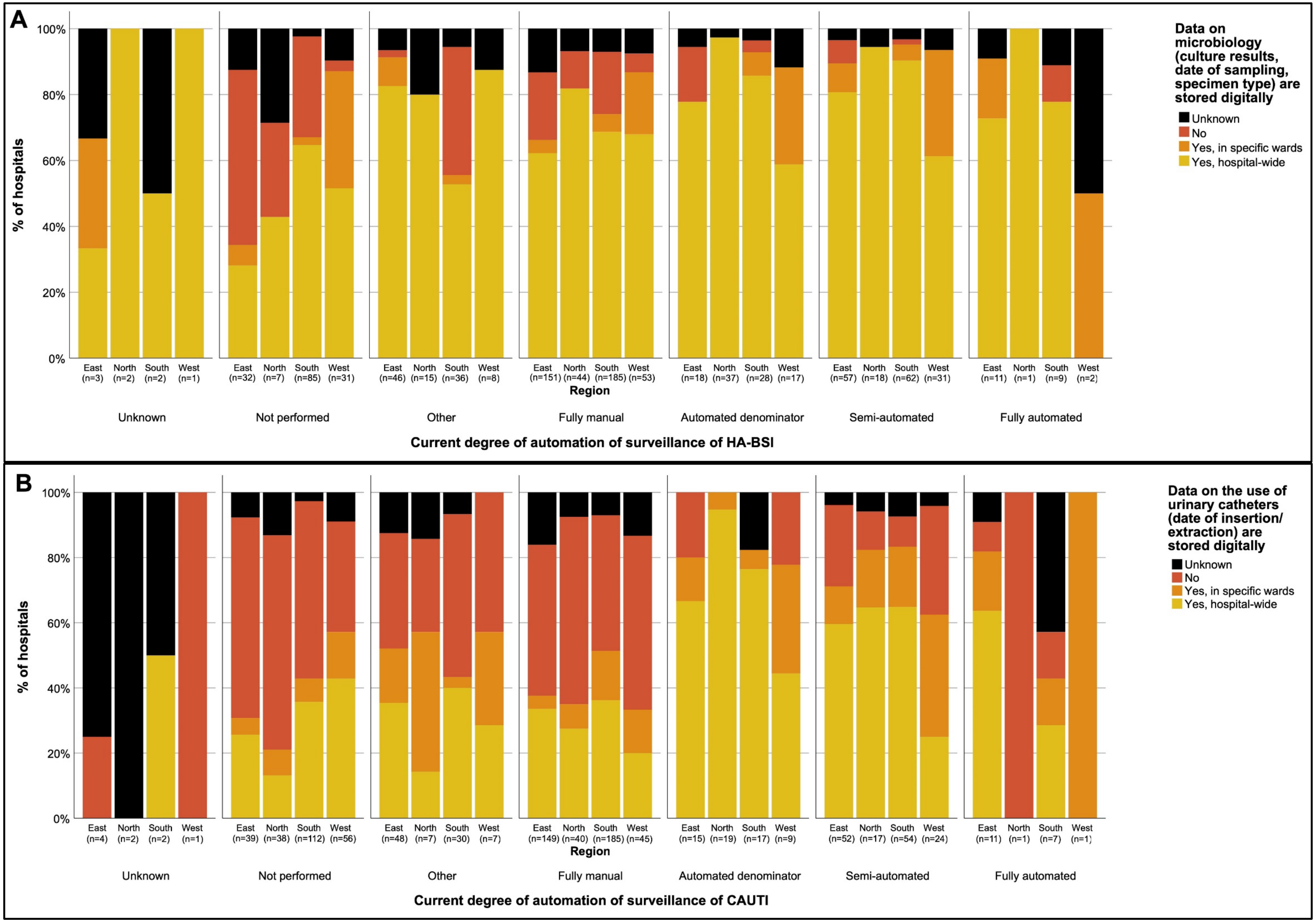

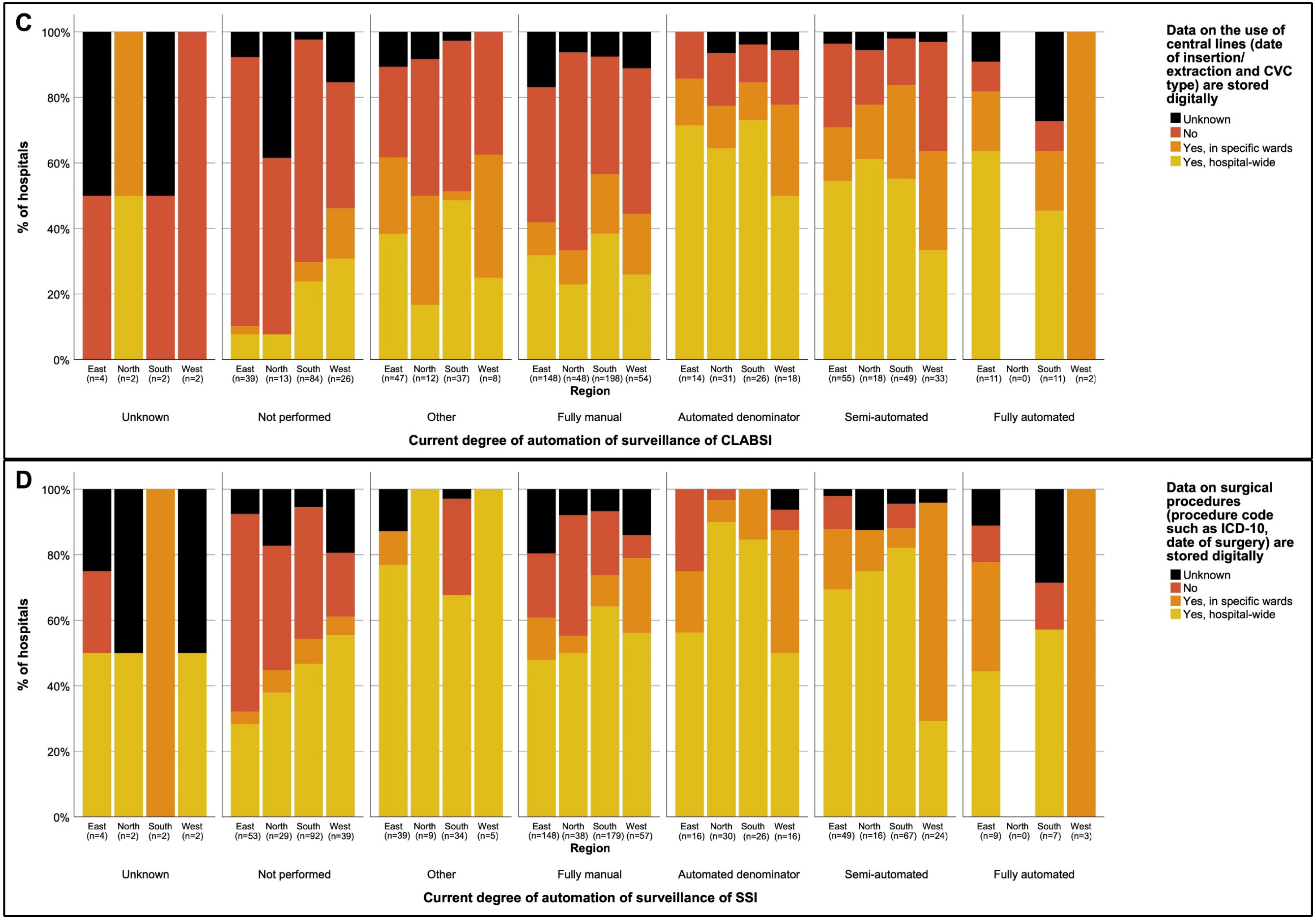

C
% of hospitals
100%
80%
60%
40%
20%
0%
East (n=4) North (n=2) South (n=2) West (n=2)
East (n=39) North (n=13) South (n=84) West (n=26)
East (n=47) North (n=12) South (n=37) West (n=8)
East (n=148) North (n=48) South (n=198) West (n=54)
East (n=14) North (n=31) South (n=26) West (n=18)
East (n=55) North (n=18) South (n=49) West (n=33)
East (n=11) North (n=0) South (n=11) West (n=2)
Region
Unknown
Not performed
Other
Fully manual
Automated denominator
Semi-automated
Fully automated
Current degree of automation of surveillance of CLABSI
Data on the use of central lines (date of insertion/ extraction and CVC type) are stored digitally
Unknown
No
Yes, in specific wards
Yes, hospital-wide
D
% of hospitals
100%
80%
60%
40%
20%
0%
East (n=4) North (n=2) South (n=2) West (n=2)
East (n=53) North (n=29) South (n=92) West (n=39)
East (n=39) North (n=9) South (n=34) West (n=5)
East (n=148) North (n=38) South (n=179) West (n=57)
East (n=16) North (n=30) South (n=26) West (n=16)
East (n=49) North (n=16) South (n=67) West (n=24)
East (n=9) North (n=0) South (n=7) West (n=3)
Region
Unknown
Not performed
Other
Fully manual
Automated denominator
Semi-automated
Fully automated
Current degree of automation of surveillance of SSI
Data on surgical procedures (procedure code such as ICD-10, date of surgery) are stored digitally
Unknown
No
Yes, in specific wards
Yes, hospital-wide

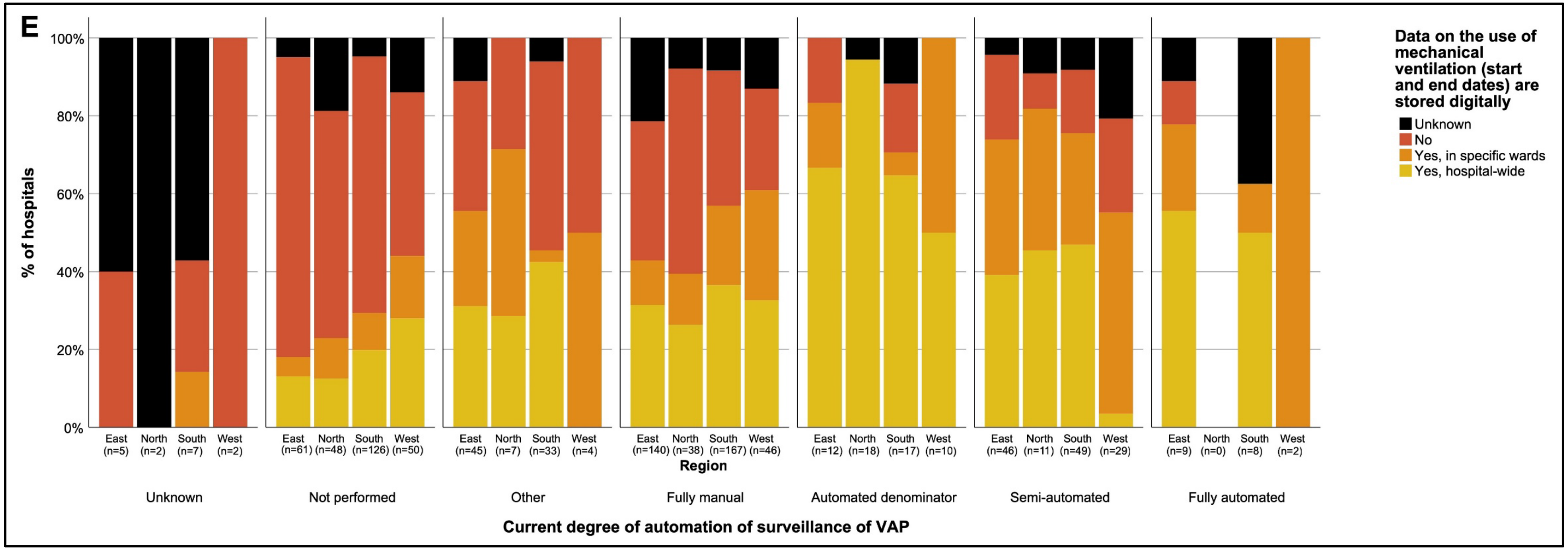

A: healthcare-associated bloodstream infection (HA-BSI), B: catheter-associated urinary tract infection (CAUTI), C: central line-associated bloodstream infection (CLABSI), D: surgical site infection (SSI), E: ventilator-associated pneumonia (VAP); Footnote: Number of hospitals are presented below the bars.
